# A multi-parameter grading system for optimal fitting of scleral contact lenses

**DOI:** 10.12688/f1000research.74638.2

**Published:** 2022-03-03

**Authors:** Preetam Kumar, Karen G. Carrasquillo, Simmy Chaudhary, Sayan Basu

**Affiliations:** 1 Bausch & Lomb Contact lens Centre, L V Prasad Eye Institute, Hyderabad, Telangana, 500034, India; 2 Bausch and Lomb School of Optometry, Brien Holden Institute of Optometry and Vision Sciences, L V Prasad Eye Institute, Hyderabad, Hyderabad, Telangana, 500034, India; 3 BostonSight, Needham, MA, 02494, USA; 4 The Cornea Institute, L V Prasad Eye Institute, Hyderabad, Hyderabad, Telangana, 500034, India

**Keywords:** Scleral contact lens, Prosthetic Replacement of Ocular Surface Ecosystem, PROSE, Contact Lens Fitting, Scleral Contact lens Fitting

## Abstract

**Background: **While scleral lens practise has improved over the years due to factors such as availability of lenses with better materials and designs as well as experience of practitioners, a lack of objectivity appears to remain in terms of assessment of scleral lens fitting. This prospective observational work aimed to achieve standardization on this front through proposing a grading system for scleral lens fitting.

**Methods: **After application of prosthetic replacement of ocular surface ecosystem (PROSE) devices on the participants’ eyes, four fundamental components for understanding scleral lens fitting such as central and limbal corneal clearance, mid-haptic compression, and alignment of lens edge over anterior sclera were assessed through a series of slit-lamp biomicroscopy imaging as well as with anterior segment optical coherence tomography. FitConnect® was used to modify the device parameters to simulate different grading patterns on the proposed scale. Serial imaging was done for all the different lenses to compose the grading scale.

**Results: **A clinically relevant grading scale was constructed that pictorially demonstrated grades for the different aspect of scleral lens fitting. The grades were conveniently scaled within three categories: “optimal”, “acceptable” and “not acceptable”.

**Conclusion: **The gradation of scleral lens fitting parameters would take a step towards objectifying the assessment patterns in practise. This will also help reducing the gap between a novice and an experienced practitioner in terms of understanding of scleral lens fitting.

## Introduction

Scleral lenses are large diameter gas permeable lenses that are usually defined by their extent of coverage over the ocular surface. Typically having a total lens diameter ranging from 16 to 24 mm, these lenses are designed to vault the entire cornea and rest on the anterior scleral surface ^1^. Since inception, scleral lenses have undergone drastic changes in their material as well as design. The introduction of gas permeable scleral lenses in 1983 by Ezekiel has been proven as a major breakthrough in terms of practise of these lenses in the field of ophthalmology ^2^. However, the popularity of these lenses has accelerated since the turn of the 21 ^st^ century due to a steady improvement in the design of the lens, as well as its ability to provide an alternative treatment option for corneal conditions which would otherwise be subjected to complicated surgical intervention ^3^. A recent review by Allen and colleagues has comprehensively demonstrated the extended role of scleral lenses in the medical community ^4^.

Reports have suggested that scleral lenses do not only help in visual rehabilitation for highly aberrated eyes, but also due to the entrapped fluid reservoir beneath the lens, help support and maintain the integrity of the ocular surface, and provide therapeutic measures for the cornea ^5– 10^.

For the most part, fitting assessment of these lenses is subjective in nature and often lacks a general consensus among practitioners ^11^. A report by Visser *et al.* has shown a systematic approach in this direction ^12^. However, the recommendation from different contact lens manufacturers, as well as from the practitioners, still varies considerably; which could especially become confusing for an emerging practitioner.

Similar challenges are faced in the field of research, when carrying out studies with and about these lenses. While the practise of these lenses will thrive on how well we understand the working principle of them, it is practically not possible to establish objective and standardized research outcomes without a robust assessment process of lens fitting. In other words, to elicit the effect of scleral lenses, we need to have a better understanding and control over the lens fitting aspect.

This work is aimed at providing an objective outlook for some of the essential parameters of scleral lens fitting assessment.

## Methods

### Instruments and devices

In this prospective observational study, conducted between 1 ^st^ April to 31 ^st^ October 2019, four post graduate students from L V Prasad Eye Institute were chosen as volunteers. The only exclusion criteria were subjects who showed unwillingness for participation. The study adhered to the tenets of Declaration of Helsinki and it was approved by the Institutional Review Board of the L V Prasad Eye Institute, Hyderabad, India (Ethics Ref LEC 07–17–049). All subjects participated after signing a written informed consent form.

**Figure 1.**
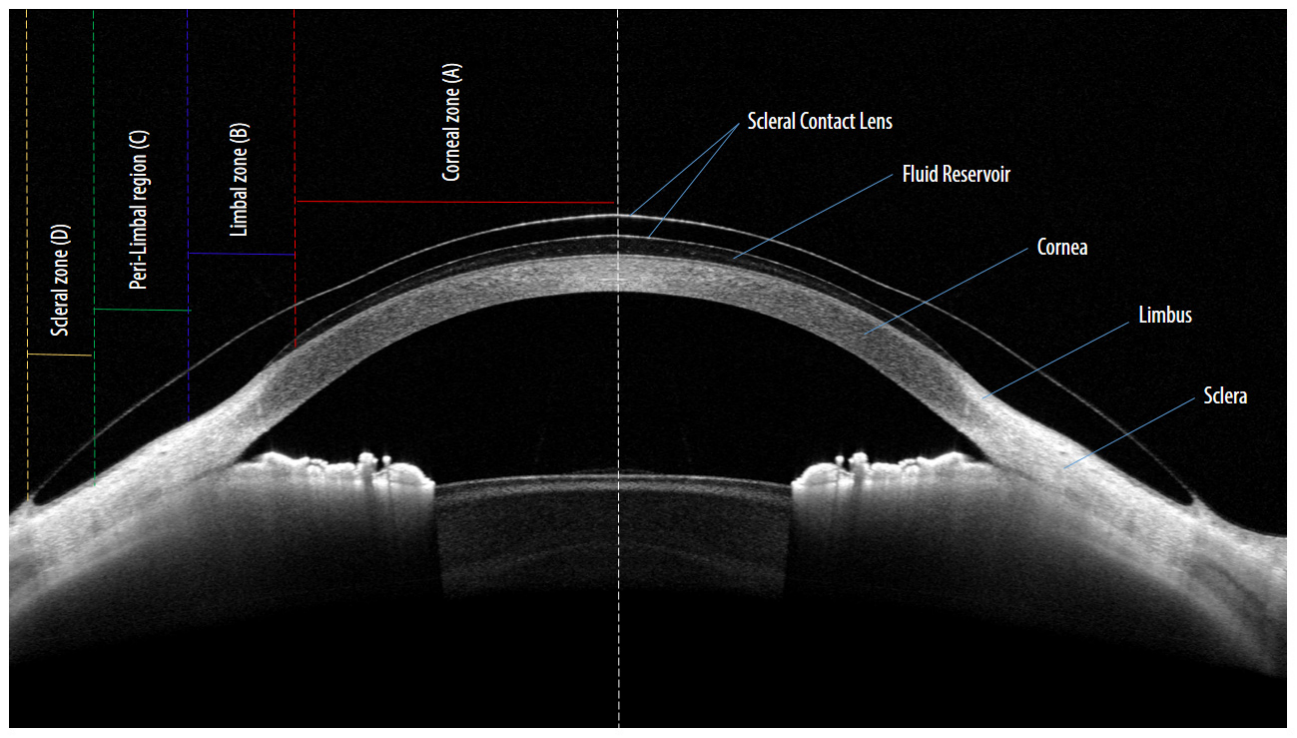
Basic parameters while fitting scleral contact lenses: Four basic lens parameters and the corresponding ocular zones while fitting scleral contact lens on the eye. Zone **A** indicate central lens vault at the centre of the corneal region (red). Zone **B** indicate Limbal lens vault within the Limbal region (blue). Zone **C** indicate mid-haptic region of the lens within the peri-Limbal region (green). Zone **D** indicate lens haptic and edge resting on the scleral region (yellow).

The prosthetic replacement of ocular surface ecosystem (PROSE) device was used for this purpose ^5^. Some features such as the fluorosilicone-acrylate material (Equalens II, Polymer Technology Corporation, Bausch & Lomb, Rochester, New York, USA), lens diameter (18.50 mm) and central lens thickness (0.3 mm) were kept constant for all the devices used in this study. The first diagnostic trial lens was selected after a thorough assessment of the cornea, surrounding scleral and the overlying bulbar conjunctiva. The overall epithelial integrity of the ocular surface, tear film stability and ocular sagittal height was noted prior to the insertion of the trial lens. Figure 1 demonstrates the important lens parameters and their corresponding zones on the ocular surface. After the documentation of lens fitting aspects in detail, a proprietary software system, FitConnect ^®^ (BostonSight, Needham, MA) was used to design lenses with modified parameters to simulate different grading scales for the individual parameters intended to evaluate. Through this software, spline mathematical function was manipulated to manoeuvre several aspects of lens design (BostonSight, Needham, MA).

Upon receiving the modified lenses, they were placed on the volunteer’s eyes. The lenses were allowed to be settled on the ocular surface for a period of one hour before commencing with the lens fit assessment. A series of slit lamp images as well as corresponding cross-sectional images with anterior segment optical coherence tomography (OCT) were captured with the lenses in place. All the slit lamp imaging was performed using the slit lamp camera (Haag Streit BM-900®). Yellow light was used for imaging and LED was used for background illumination for enhancement of image quality. Typically, a 10X magnification of the observation system and an 8 mm aperture height of the illumination system was kept constant for all the images. Once, the slit lamp images were deemed satisfactory, they were backed by corresponding anterior segment OCT images. OptoVue XR Avanti® (Fremont, California, USA) was used for this purpose. Avanti OCT provides 70,000 A-scans with an axial resolution of 5 microns and a scan length of 8 mm on the cornea. For measurement of central and limbal lens vault, volunteers were advised to fixate at the centre and a “cross-line” setting was used to capture the image. For the assessment of lens impingement at the lens periphery, a “single-line” setting was used to capture the image and volunteers were asked to maintain a down gaze to get the full extent of the lens periphery.

## Results

### Scleral lens fitting: grading system

The fundamental criteria for scleral lenses are to vault the cornea completely at all areas. However, there are other parameters as well that are critical in a successful lens fitting. Central lens clearance, limbal clearance, lens compression at the mid haptic area, lens impingement and edge lift at the periphery, are five important parameters that form the basis of scleral lens fitting and its assessment. For this reason, these parameters were chosen to be featured in the form of grading scales in our report. In this process, slit lamp images along with corresponding anterior segment (AS)-OCT images were captured for documentation.

**Figure 2.**
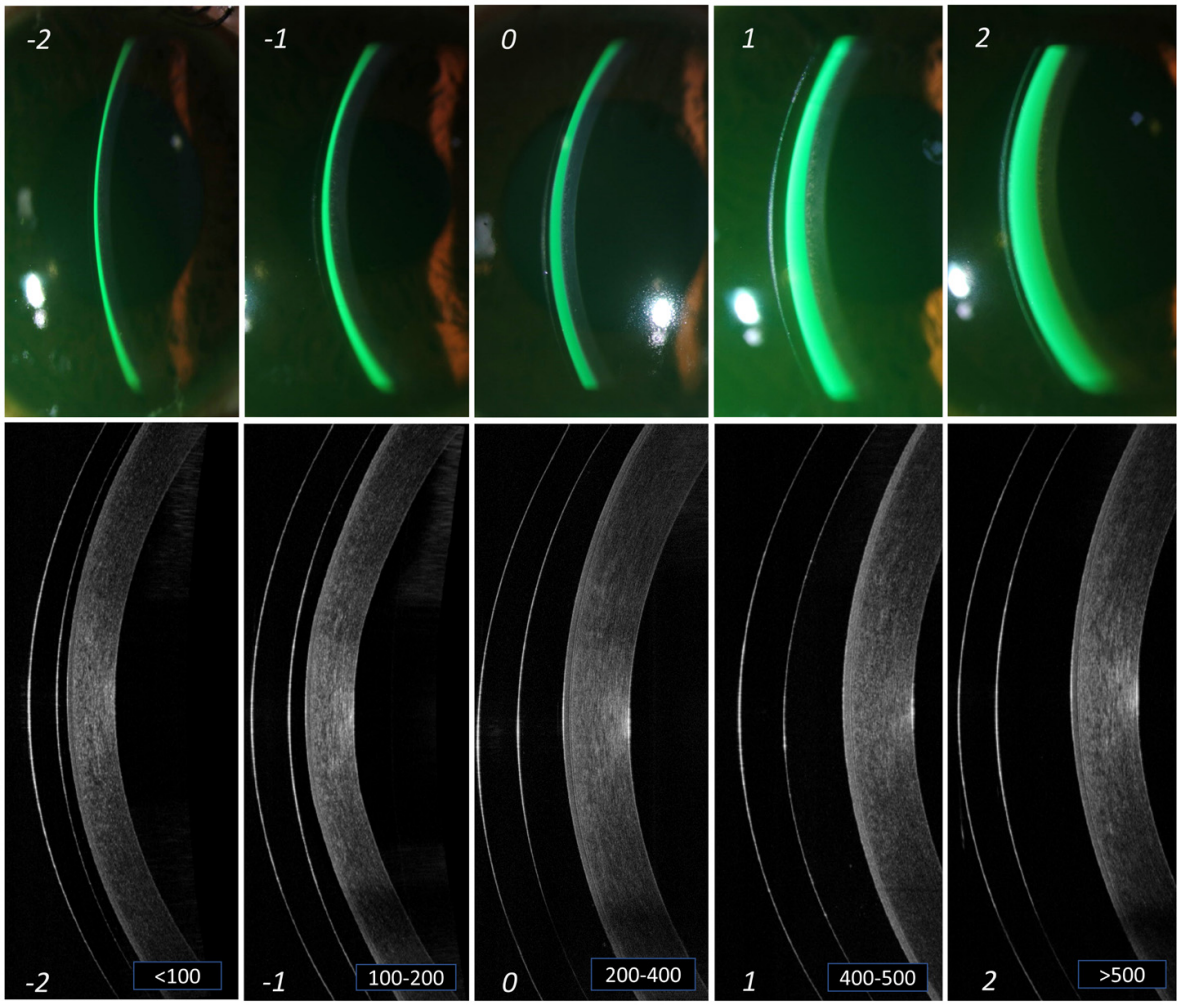
Grading of central lens vault. Different grades of lens vault noted at the centre of cornea from slit lamp imaging (a) and AS-OCT imaging (b). The thickness of the tear reservoir (marked in green after instillation of sodium fluorescein) indicates the magnitude of lens vault. From left to right (panels A to E) are arranged in an ascending order of lens vault. The corresponding grades are mentioned at the bottom of each panel. Grade '0' in the middle (panel C) indicates an optimum central lens vault. Grade -1 and +1 (panels B and D) indicates central vault still within "acceptable" range. However, grade -2 and +2 (panels A and E) indicates central vault that is "not acceptable" and needs immediate attention for modification.

***Central corneal clearance.*** The space between the anterior surface of the cornea and the posterior surface of the lens at the centre of the cornea is defined as the central corneal clearance. Assessment of this parameter is extremely important as this primarily helps in avoiding any mechanical induced complications of the cornea. Furthermore, the saline that fills up this space maintains the hydration of the corneal surface. Practitioners need to be careful while assessing this parameter, as reports suggest that the aforementioned space tends to decrease over time during lens wear due to the pliable nature of the bulbar conjunctiva, over which the lenses settle ^13^. In a clinical setting, the central clearance reservoir is best observed with instillation of sodium fluorescein (NaFl) into the fluid reservoir. The fluorescence of NaFl allows to clearly demarcate areas of clearance by showcasing a green space between the lens and the corneal surface, while a black appearance denotes areas of touch or contact between those two surfaces. Additionally, the intensity of NaFl fluorescence (green appearance) can further highlight the magnitude of the space between these surfaces (i.e., an increased clearance would show a higher intensity of fluorescence (green colour) compared to the areas of lesser clearance). However, a cross section view with the help of the optic section illumination system of slit lamp bio microscope would further help in rough estimation of the space underneath the lens, using the known lens centre thickness as reference. Table 1 and Figure 2 indicates the different grades of central corneal clearance, where the “grade 0”, i.e., space between 200 to 400 microns appears to be the criteria for considering that lens fit to be at an “optimal” level. Grade -1 and +1 can be considered at an “acceptable” level, but not without monitoring, while Grade -2 and +2 are considered at a “not acceptable” level.

**Table 1.** Grading to assess the different parameters of scleral lens fitting. Recommended grading of the fundamental parameters for the assessment of scleral contact lens fitting.

Parameter	Grading scale						
	-3 Not Acceptable	-2 Not Acceptable	-1 Acceptable	0 Optimal	+1 Acceptable	+2 Not acceptable	+3 Not Acceptable
Central clearance (µ)	N/A	<100	100 - 200	200 - 400	400 - 500	>500	N/A
Limbal clearance (µ)	N/A	<50	50 - 100	100 - 200	200 - 300	>300	N/A
Mid-haptic compression	N/A	N/A	N/A	No difference in tissue colour at the mid haptic area of the lens Or, No obstruction of minor and major vessels	Mild difference in tissue colour at the mid haptic area of the lens Or, Obstruction of minor vessels but no obstruction of major vessels	Moderate difference in tissue colour and appearance of “white band” at the mid haptic area of the lens Or, Obstruction of minor vessels and obstruction of ≤2 major vessels	Severe difference in tissue colour and appearance of a prominent “white band” at the mid haptic area of the lens Or, Obstruction of minor vessels and obstruction of >2 major vessels
Edge alignment	Severe EL * On optic section, prominent split between reflections inside and outside the lens edge along with entrapped free-flowing air bubble	Moderate EL * On optic section, moderate split between reflection inside and outside the lens edge, does not necessarily associate with air bubble entrapment	Mild EL * On optic section, mild split between reflection inside and outside the lens edge with no air bubble entrapment	Optimal Well aligned lens edge. i.e. On optic section, reflex outside the lens margin maintains alignment with the reflex inside the margin and does not show any split or break	Mild IMPGT ^€^ On optic section, reflex outside the lens margin is mildly more curved compared to the reflex inside the lens margin	Moderate IMPGT ^€^ On optic section, reflex outside the lens margin is moderately more curved compared to the reflex inside the lens margin (blanching of blood vessels expected)	Severe IMPGT ^€^ On optic section, reflex outside the lens margin is visibly more curved compared to the reflex inside the lens margin (obvious blanching of blood vessels expected

(*EL: lens edge lift; ^€^IMPGT: lens impingement)

**Figure 3.**
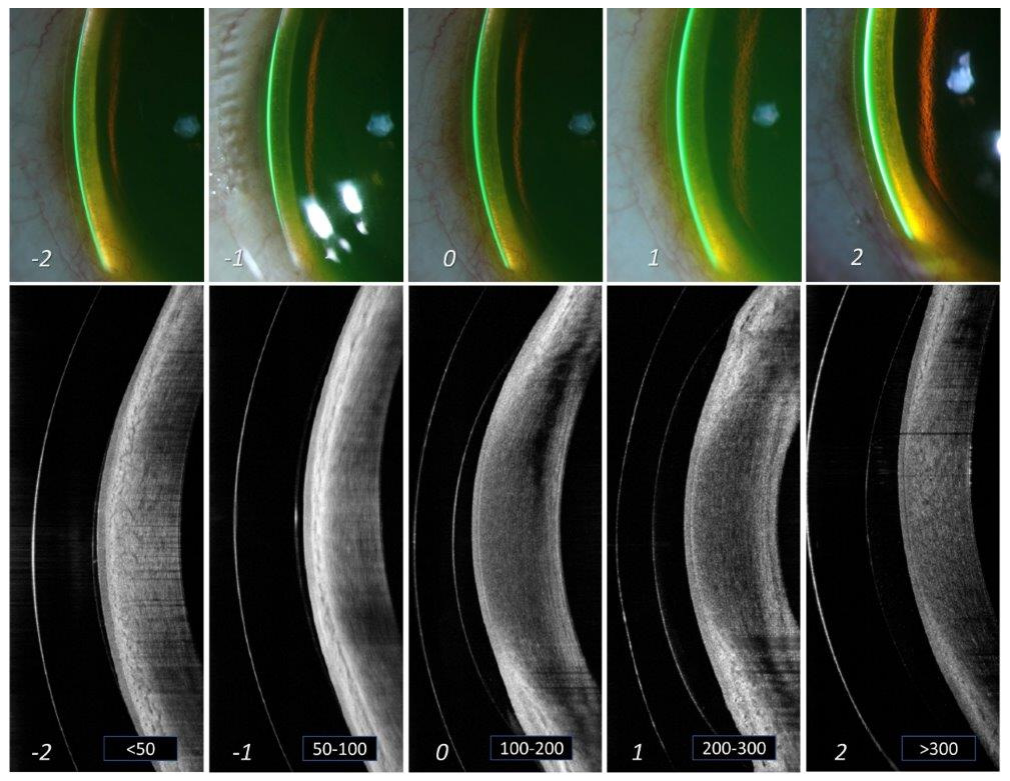
Grading of limbal lens vault. Different grades of lens vault noted at the limbus of cornea from slit lamp imaging (a) and AS-OCT imaging (b). The thickness of the tear reservoir (marked in green after instillation of sodium fluorescein) indicates the magnitude of lens vault. From left to right (panels A to E) are arranged in an ascending order of lens vault. The corresponding grades are mentioned at the bottom of each panel. Grade '0' in the middle (panel C) indicates an optimum lens vault at limbus. Grade -1 and +1 (panels B and D) indicates limbal vault still within "acceptable" range. However, grade -2 and +2 (panels A and E) indicates limbal vault that is "not acceptable" and needs immediate attention for modification.

***Limbal clearance.*** The aforementioned methods of assessment of corneal clearance holds true for the limbus as well. Assessment of this parameter is important for understanding the amount of mechanical interaction of the lens at the limbal area which hosts the stem cells that are crucial for maintenance of corneal health. Table 1 and Figure 3 shows the different grades of limbal corneal clearance and its correlation to amount of clearance in microns, where “grade 0”, i.e., space between 100 to 200 microns appears to be the criteria for considering that lens fit to be at an “optimal” level. Grade -1 and +1 can be considered at an “acceptable” level, but monitoring is warranted. Grade -2 and +2 are considered at a “not acceptable” level. Similar to the corneal measurement, the lens thickness was taken as a reference to which, limbal clearance was measured. Maximum effort was directed initially, towards procuring all the slit lamp images at the same area near the limbus ( Figure 3).

**Figure 4.**
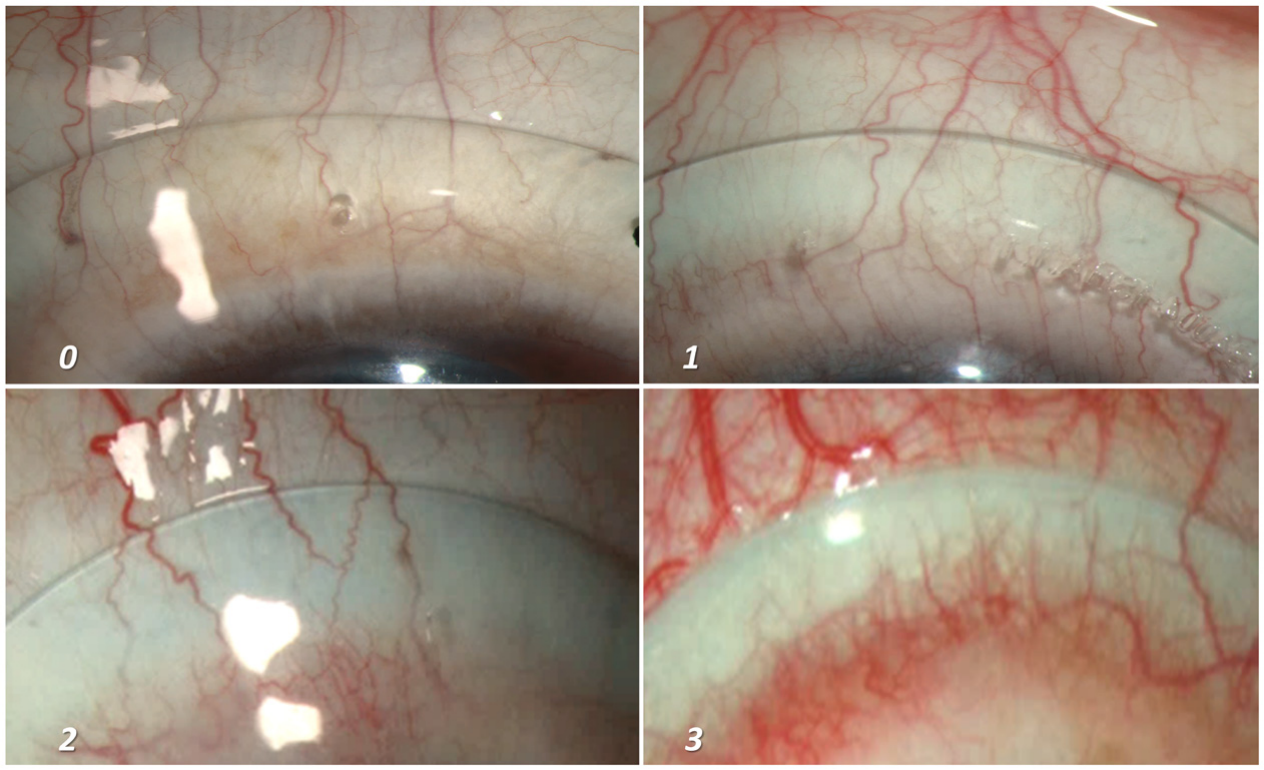
Grading of mid-haptic compression. Different grades of mid-haptic compression on conjunctival tissue and blood vessels. The intensity of the “whitish band” at the mid haptic area in comparison to the adjacent tissue colour along with the potential occlusion of the capillaries (minor vessels) as well as major vessels of the bulbar conjunctiva marks the intensity of mid-haptic compression. Panel A indicates an optimum scenario where neither the major vessels nor the capillaries are compressed by the mid haptic region of the lens. In addition, the tissue colour of the underlying bulbar conjunctiva remains indistinguishable. Panel B depicts the lens fitting where we start observing the difference in the tissue colour around the mid haptic area due to the compression at that area. However, this is considered as “acceptable” as the major conjunctival vessels remain unaffected. Panels C and D demonstrates an increasing difference in tissue colour and an appearance of a prominent “white band” at the mid haptic area of the lens. An additional impact on major vessels makes the lens fit “not acceptable” and needs immediate attention for modification.

***Mid haptic compression.*** The lens haptic supports the weight of the lens as the vaulted corneal space lands on the overlying conjunctival tissue and blood vessels. A larger haptic area promotes and ensures a larger surface area to allow for even and homogeneous lens weight distribution and minimize compression on the conjunctival vessels. Excess lens pressure on the conjunctival vessels will cause blockage in the blood flow, giving the appearance of blanched or whitened focal areas underneath the lens haptic, while the lens is being worn, a phenomenon that is usually termed as “blanching”. A lens fit that exhibits significant conjunctival blanching will manifest signs of rebound hyperaemia on the bulbar conjunctiva upon lens removal and is often associated with eye pain and tenderness that can linger for few hours to sometimes days, depending on the amount of haptic compression and duration of lens wear. Table 1 and Figure 4 shows the different grades of mid haptic compression, where “grade 0” indicates an optimal situation where neither the minor vessels (capillaries) nor the major blood vessels get compressed by the lens haptic. Grade 1 indicates compression of minor vessels by the mid haptic area. Although, the major vessels remain unaffected and can be considered within acceptable range. However, a lens which shows Grade 2 or 3 compressions (blanching of all the minor vessels along with the blanching of major vessels) will usually require a reconsideration regarding the lens fitting. Our volunteers’ eyes were usually devoid of any significant bulbar conjunctival congestion with absence of prominent blood vessels. Hence, for the purpose of portraying the different grades of mid-haptic compression, patients’ eyes (that already had existing bulbar conjunctival congestion) were recruited for photography ( Figure 4). The quadrant that was most prominent to show the effect of lens compression was chosen for the photography. One of the ways to describe the mid haptic compression is the intensity of appearance of the “whitish” band, specifically in contrast with the adjacent conjunctival tissue colour at the region of lens mid haptic, which shows the level of congestion in the anterior blood vessels. Figure 4 provides a demonstration of this as an increase in intensity was quite apparent as we moved from grade “0” to grade “3”.

**Figure 5.**
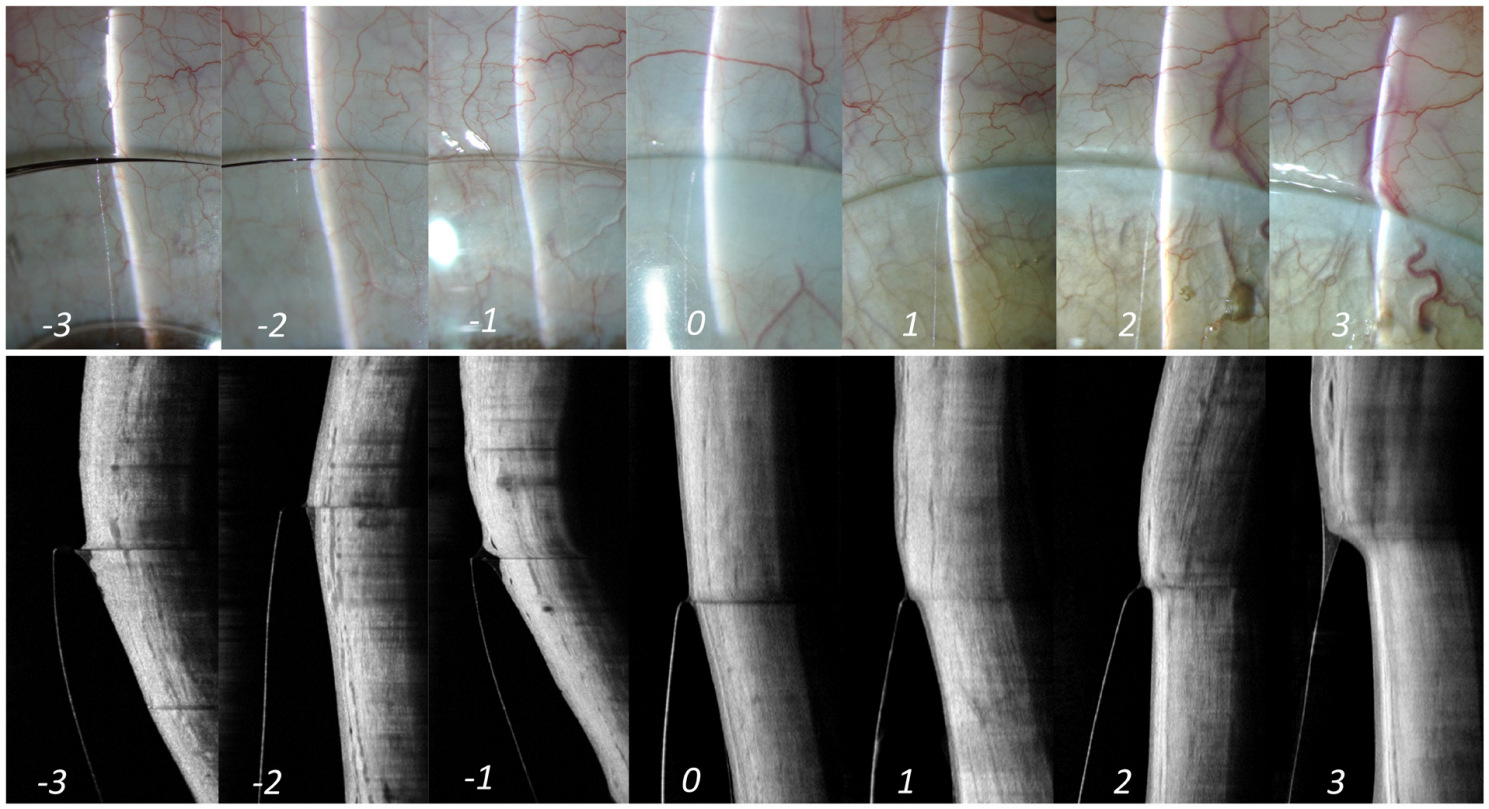
Edge alignment. Illustration of lens alignment over the anterior sclera from slit lamp (top panel) as well as AS-OCT (bottom panel) imaging. The corresponding grades are mentioned at the bottom of each panel. Grade '0' in the middle indicates an optimum alignment of lens. Grade -1 and +1 indicates an alignment of edge within "acceptable" range. However, grades -2 and +2 or grades -3 and +3 indicates alignment of lens edge that is "not acceptable" and needs immediate attention for modification.

***Edge alignment.*** This parameter describes the landing relationship between lens edge and the underlying conjunctival tissue. The proper assessment of lens edge to bulbar conjunctiva relationship allows practitioners to determine whether the lens periphery is exhibiting a “sink-in” or “impinging” effect or a “lift-off” or “edge lift” effect over the conjunctival surface. Different techniques have been postulated and are interchangeably used in practice. Some practices attempt to assess the fluid exchange through the lens edge with the application of fluorescein and assessing its passage underneath the lens. The longer it takes for the fluorescein to reach the centre/optic portion of the lens, indicates a tighter lens edge on the ocular surface. Conversely, the faster it takes for the same, indicates a lens edge that is more lifted from the ocular surface. Some reports have suggested that OCT can be a useful tool to assess the lens edge profile and its settlement over the ocular surface ^13^. However, in a clinical setting, this could be easily observed under slit-lamp evaluation. An optic section illumination technique compared to a diffuse illumination helps in better understand and assess this parameter. A line scan from the anterior segment OCT imaging can further enhance the view of the same. When an optic section of slit lamp or a line scan from AS-OCT is aligned over the lens edge, there are essentially two areas of the slit that requires close observation, one that is within the lens edge (i.e., linear reflection inside the lens) and the other that is just beyond the edge (reflection outside the lens) ( Figure 5). A perfectly aligned reflection (i.e., reflection inside and outside the lens shows no split and appears as a straight line in slit lamp as well as AS-OCT image) indicates an aligned edge (Grade 0, Figure 5), whereas a deviation from a straight-line appearance or a split between these two sections indicates either an impingement or a lift of the lens edge.

When the reflection outside the lens (conjunctival region) appears more curved compared to the reflection within the lens, contrary to the straight-line appearance as described in the alignment fit, indicates lens impingement. A positive numbering in the grading scale refers to impingement, as it indicates greater contact with the conjunctival surface overlying the sclera (Grading 1 to 3, Figure 5). Conversely, presence of an evident split between the two zones of the reflection across the lens edge indicate edge lift (lens haptic is lifted off the ocular surface). A negative numbering in the grading scale refers to edge lift, as it indicates less contact with the conjunctival surface overlying the sclera (Grading -1 to -3, Figure 5). Table 1 shows the different grades of lens impingement and edge lift. Herein,” grade 0” indicates an “optimal” alignment, indicating a smooth settlement of the lens periphery over the ocular surface. Grade -1/+1 are graded at an “acceptable” level. Grade -2/+2 and -3/+3 are graded at a “not acceptable” level.

While a moderate state of either impingement or edge lift (Grade -2 and +2) may not necessarily cause a concern immediately after lens insertion, this could possibly become an issue either in the form of physiological changes in the ocular surface or in terms of vision on a long run with the usage of these lenses; requiring the modification of the lens parameters accordingly. However, a severe state of the same (Grade -3 and +3) needs immediate attention and requires a significant modification in all cases.

## Discussion

This report aimed at documenting the important features considered during the fitting of scleral lenses. This exercise was, in large part, instigated from the dilemma that we often experienced during our clinical practice, both during intra-person assessment of same lens fitting in separate sessions as well as inter-person assessment of same lens fitting during a single session. This circumstance was quite aptly supported by the recent report from the SCOPE study group, distinctly illustrating a sense of ambiguity between practitioners, both in terms of selection as well as assessment of scleral lens parameters ^11^. The rate of complications after scleral lens wear has decreased over the years mainly due to a significant improvement in contact lens materials and designs. However, there are other aspects that can contribute to further enhance the success of contact lens practise. Having a consensus on scleral lens fitting assessment and the way we refer to fitting end points is extremely important for the standardization of its practise and possibly will serve as a step in that direction.

Central lens clearance has long been speculated as a causative factor for a reduction in the amount of oxygen reaching the underlying corneal surface. Recent work by Harthan *et al.* has clearly demonstrated the lack of harmony in terms of selection of scleral lens vault ^11^. A report by Compan and colleagues showed the difference in corneal swelling in mini scleral lens user (15.5 mm) having a shallower post lens tear thickness (1.6%), vis-à-vis, deeper reservoir (3.9%) ^14^. In this report, an 18.50 mm lens diameter was used. With the PROSE device in particular, we can customize the clearance to any specified amount, regardless of lens diameter. Although, the magnitude of swelling found in these studies falls within the physiological limit in the presence of closed eye conditions, this is still considered as one of the prime parameters when evaluating a scleral lens fit. Therefore, in clinical practice and in research, contact lens practitioners need to have a proper and standardized assessment tool in place. The role of AS-OCT has often been mentioned in the literature for the quantification of the space behind the lens. However, with the rapid expansion and adoption of scleral lenses across the entire spectrum of eye care (optometry and ophthalmology), it is easy to assume that not all practices would possess the instrument, hence lens assessments with basic instruments, such as a slit lamp bio microscope remains fundamental in practice.

Similar to central clearance, evaluation of the limbal area is considered an equally important component of scleral lens fitting assessment. A confirmation of clearance at the limbal area is required to minimize the pressure on this critical area known to store stem cells that promote epithelial cell regeneration and help ensure corneal health. The adverse impact of scleral lens pronounced touch and heavy indentation at the limbal area is well documented by Walker and colleagues in their report ^15^. In unavoidable circumstances, it is cautioned and advised to have a maximum of 20% touch of the overall circumference of the limbus. However, the design of a lens also plays a major role in avoiding a limbal lens touch and the PROSE device used in this report, is a design customized using a software powered by spline technology, allowing an extremely efficient customization of every lens parameter, hence benefitting in these circumstances ^16^.

A tightly fit scleral lens, typically termed as a lens “seal-off” is considered a significant issue concerning scleral users. Primarily, this requires a higher mechanical effort to dislodge the lens from its position, which can potentially cause ocular micro trauma. In addition, this situation would possibly promote a stagnation of metabolic waste rich fluid which could result in toxic response of the corneal epithelial tissue causing inflammatory events as mentioned by Sverinsky and colleagues ^17^. As cited previously, the report by Harthan *et al.* has clearly mentioned the difference in opinion between the novice and experienced practitioners while deciding upon the final lens in terms of blanching of blood vessels at the mid peripheral region of the lens ^11^. This study aimed to create a platform to easily understand the magnitude of blanching at any given quadrant. A diseased population was chosen to represent this grading as it was difficult to simulate the desired patterns in the normally white eyes (with minimum prominent blood vessels on the surface).

The transition from the corneo-scleral junction to the anterior sclera is physiologically asymmetric ^18, 19^ and often translates the same when it comes to settlement of scleral lens periphery onto the ocular surface. While a loosely fit lens will show sign of areas of the lens elevated from the ocular surface, a tightly fit lens would show indentation on the malleable bulbar conjunctival tissue. An elevated lens edge would allow more debris from the ocular surface to get into the fluid reservoir underneath the lens causing visual disturbances. Moreover, a significant edge lift would result in the post lens fluid to seep out of the lens periphery causing air bubble to form which may have an immediate effect on the visual acuity ^1^ and potentially lead to dellen formation. On the other hand, a lens periphery that digs deep into the conjunctival tissue can cause a significant reduction of tear exchange underneath the lens, it can potentially reduce the average wearing time of the lens usage, and more chronically it can lead to hypertrophy of the conjunctival tissue.

Visser *et al.* have previously recommended a grading scale. However, their report included findings such as air bubble, lens surface wettability, and front and back surface deposits ^12^. In our practice, we generally do not recommend any air bubble entrapment under the lens. Unless working with a fenestrated lens design, air entrapment underneath should be avoided, to promote a healthier ocular surface, as air bubbles at times can become stagnant and lead to complications from chronic desiccation or dellen formation. In practise, agreeing upon a single objective point of acceptability may not appear convenient due to factors such as chair time spent for each patient, as well as the inter- personal variability of the assessment process itself. Accordingly, in addition to recommending the “optimal” lens fitting characteristic, this report also suggests an “acceptable” territory ( Table 1) in which the practitioner can manoeuvre and dispense the contact lens for the patients.

In summary, this study looked into parameters that we consider fundamental for a successful scleral lens fitting. The advantages of this could be few folds- one, the dilemma in terms of finding the ideal scleral lens fit would decrease, lowering the chair time spent on each patient. Second, a unified perspective on lens fitting will go in some way bridging the gap between an experienced practitioner and a novice, standardizing the practise in general. Third, categorizing the lens fitting aspect and having a clear demarcation between an acceptable and an ill fit lens is important in the field of research as we can be assured of the impact of these lenses.

## Data availability

This article proposes a grading system based on observation, with the images included being representative of the grading system. There is therefore no other data or analysis.
