# Supplementary material for: A multi-parameter grading system for optimal fitting of scleral contact lenses
Source: F1000Res. 2022 Mar 3;11:6. Originally published 2022 Jan 5. [Version 2] doi: 10.12688/f1000research.74638.2 (PMC8802154; doi:10.12688/f1000research.74638.2)
Supplement: Peer review history

# Peer review: Reviewer response for version 2

**DOI:** 10.5256/f1000research.120811.r126102

**Published:** 2022-05-13

**Authors:** Sharon D'Souza

**Affiliations:** 1 Department of Cornea and Refractive Surgery, Narayana Nethralaya Eye Hospital, Bengaluru, Karnataka, India

The authors have made revisions to the article to improve it and improve the reader's understanding of the subject including the figure 1.

 The various images and illustrations are a ready reckoner for customizing scleral lens fit.

Is the work clearly and accurately presented and does it cite the current literature?

Yes

If applicable, is the statistical analysis and its interpretation appropriate?

Not applicable

Are all the source data underlying the results available to ensure full reproducibility?

Yes

Is the study design appropriate and is the work technically sound?

Yes

Are the conclusions drawn adequately supported by the results?

Yes

Are sufficient details of methods and analysis provided to allow replication by others?

Yes

Reviewer Expertise:

Cornea Ocular surface and refractive surgery

I confirm that I have read this submission and believe that I have an appropriate level of expertise to confirm that it is of an acceptable scientific standard.

# Peer review: Reviewer response for version 2

**DOI:** 10.5256/f1000research.120811.r126101

**Published:** 2022-03-03

**Authors:** Sotiria Palioura

**Affiliations:** 1 Department of Ophthalmology, Bascom Palmer Eye Institute, Miller School of Medicine, University of Miami, Miami, FL, USA

No comments.

Is the work clearly and accurately presented and does it cite the current literature?

Yes

If applicable, is the statistical analysis and its interpretation appropriate?

Not applicable

Are all the source data underlying the results available to ensure full reproducibility?

Yes

Is the study design appropriate and is the work technically sound?

Yes

Are the conclusions drawn adequately supported by the results?

Yes

Are sufficient details of methods and analysis provided to allow replication by others?

Yes

Reviewer Expertise:

Cornea, Ocular Surface, Cornea Transplantation, Conjunctival Tumors, Cataract Surgery

I confirm that I have read this submission and believe that I have an appropriate level of expertise to confirm that it is of an acceptable scientific standard.

# Peer review: Reviewer response for version 1

**DOI:** 10.5256/f1000research.78413.r118751

**Published:** 2022-01-28

**Authors:** Sharon D'Souza

**Affiliations:** 1 Department of Cornea and Refractive Surgery, Narayana Nethralaya Eye Hospital, Bengaluru, Karnataka, India

An excellent paper with lucid take home points and clinical relevance for all practitioners of scleral contact lens. By giving a clear step by step methodology for scleral lens fitting, the authors have allowed for better reproducibility of good scleral contact lens fitting results. This is extremely important to avoid long term challenges and complications of poorly fit lenses. They have clearly listed the 4 parameters to be evaluated while doing a scleral lens fitting and provided a range of ‘acceptable territory’ rather than a single point of accurate fit to allow for variability between practitioners and practices. The clinical slit lamp images and anterior segment OCT images add a lot of clarity to each step and can be used as a ready reckoner while doing the contact lens fitting. By grading the contact lens fit into optimal, acceptable and not acceptable, supported by clinical images, they very clearly elucidate when immediate action must be taken to avoid complications. In addition to explaining the features of an ideal fit, the authors also clearly explain the possible issues which can occur if the patient continues to use a poor fit. The article also highlights the advantages of the customizable PROSE contact lenses.

Minor revisions

- The authors can consider adding in a line diagram of a scleral contact lens and how it fits on the cornea and sclera with the important parts highlighted to further improve the understanding for the beginner user.
- Figure 4 beautifully depicts the edge alignment on the sclera. Adding annotations or arrows at areas of interest may further simplify the understanding for the reader. The same can be applied to other figures if required.
- The methodology of assessment of lens fit on ASOCT has been explained very well and will be useful for clinicians to reproduce in their practice.
- Please mention whether the assessment of contact lens fit was done immediately after insertion or after a gap as these lenses are known to settle over time. The ideal time for assessment can also be mentioned.
- Paragraph 2 - The first diagnostic trial lens was selected after a thorough assessment of the cornea, surrounding scleral and the overlying bulbar conjunctiva.
- Can mention what parameters of the cornea and sclera were assessed.
- The summary highlights the important take home points from the paper.
- A few typographical errors through the manuscript to be corrected.

Introduction paragraph 2 - While the practice of these lenses will thrive on how well we understand the working principle of them, is practically not possible to establish objective and standardized research outcomes without a robust assessment process of lens fitting

- Change to "...principle of them, *it* is practically..."

Methods paragraph 2 - such as the fluorosilicone-acrylate material (Equalens II, Polymer Technology Corporation, Bausch & Lomb, Rochester, New York, USA), lens diameter (18.50 mm) and central lens thickness (0.3 mm) was kept constant for all the devices used in this study.

- Change was to *were*

Page 5 paragraph 2 - Excess lens pressure on the conjunctival vessels will cause blockage in the blood flow, given the appearance of blanched or whitened focal areas underneath the lens haptic, while lens is being worn, a phenomenon that is usually termed as “blanching”.

- Change given to *giving*

Is the work clearly and accurately presented and does it cite the current literature?

Yes

If applicable, is the statistical analysis and its interpretation appropriate?

Not applicable

Are all the source data underlying the results available to ensure full reproducibility?

Yes

Is the study design appropriate and is the work technically sound?

Yes

Are the conclusions drawn adequately supported by the results?

Yes

Are sufficient details of methods and analysis provided to allow replication by others?

Yes

Reviewer Expertise:

Cornea Ocular surface and refractive surgery

I confirm that I have read this submission and believe that I have an appropriate level of expertise to confirm that it is of an acceptable scientific standard.

# Response

**Published:** 2022-02-19

**Authors:** Sayan Basu

**Affiliations:** L V Prasad Eye Institute, Hyderabad, India

**Comment 1**: The authors can consider adding in a line diagram of a scleral contact lens and how it fits on the cornea and sclera with the important parts highlighted to further improve the understanding for the beginner user.

***Response***: Thank you for your suggestion. A new figure has been added (figure 1) in the manuscript. This figure demonstrates a cross sectional image of the eye with a scleral contact lens in place, highlighting the important lens parameters and their corresponding ocular zones. The sequence of the other figures in the manuscript has been modified accordingly.

**Comment 2**: Figure 4 beautifully depicts the edge alignment on the sclera. Adding annotations or arrows at areas of interest may further simplify the understanding for the reader. The same can be applied to other figures if required.

***Response***: Thank you for the suggestion. We believe that in its present form, the interpretation of the image is quite intuitive and since it is a composite of 14 images, adding more annotation may make it look quite cluttered.

**Comment 3**: The methodology of assessment of lens fit on ASOCT has been explained very well and will be useful for clinicians to reproduce in their practice.

***Response***: Thank you for your comment.

**Comment 4**: Please mention whether the assessment of contact lens fit was done immediately after insertion or after a gap as these lenses are known to settle over time. The ideal time for assessment can also be mentioned.

***Response***: Thank you for your suggestion. This has now been mentioned under the “Methods-instruments and devices” section- third paragraph.

**Comment 5**: Paragraph 2 - The first diagnostic trial lens was selected after a thorough assessment of the cornea, surrounding scleral and the overlying bulbar conjunctiva.

 Can mention what parameters of the cornea and sclera were assessed.

***Response***: Thank you for your suggestion. This has now been mentioned under the same section- “Methods-instruments and devices” section- second paragraph.

**Comment 6**: The summary highlights the important take home points from the paper.

***Response***: Thank you for your comment.

**Comment 7**: A few typographical errors through the manuscript to be corrected.

 Introduction paragraph 2 - While the practice of these lenses will thrive on how well we understand the working principle of them, is practically not possible to establish objective and standardized research outcomes without a robust assessment process of lens fitting

 Change to "...principle of them, it is practically..."

***Response***: Thank you for your suggestion. The change has been made in the manuscript.

**Comment 8**: Methods paragraph 2 - such as the fluorosilicone-acrylate material (Equalens II, Polymer Technology Corporation, Bausch & Lomb, Rochester, New York, USA), lens diameter (18.50 mm) and central lens thickness (0.3 mm) was kept constant for all the devices used in this study.

 Change was to were

***Response***: Thank you for your suggestion. The change has been made in the manuscript.

**Comment 9**: Page 5 paragraph 2 - Excess lens pressure on the conjunctival vessels will cause blockage in the blood flow, given the appearance of blanched or whitened focal areas underneath the lens haptic, while lens is being worn, a phenomenon that is usually termed as “blanching”.

 Change given to giving

***Response***: Thank you for your suggestion. The change has been made in the manuscript.

# Peer review: Reviewer response for version 1

**DOI:** 10.5256/f1000research.78413.r118750

**Published:** 2022-01-10

**Authors:** Sotiria Palioura

**Affiliations:** 1 Department of Ophthalmology, Bascom Palmer Eye Institute, Miller School of Medicine, University of Miami, Miami, FL, USA

This work aims to standardize a much needed practice, scleral lens fitting, by providing a clinically relevant grading system. Four components were utilized towards this end: a. central conreal clearance, b. limbal clearance, c. mid-haptic compression, and d. alignment of lens edge over anterior sclera. These components were assessed through slit-lamp photography and anterior segment optical coherence tomography.

 The authors have done an excellent job in assigning well-explained and clearly illustrated parameters to the practice of scleral contact lens fitting. They should be commended for their efforts. This report makes scleral contact lens fitting more understandable even to novice practitioners and it can undoubtedly aid in evaluating the performance of scleral contact lenses from a research perspective as well.

 Minor comments:

1. Introduction – 1 ^st^ paragraph: please change “its” to “their” in the following sentence “Since inception, scleral lenses have undergone drastic changes in its material as well as design.”
2. Introduction – 1 ^st^ paragraph: please change “have” to “has” in the following sentence “However, the popularity of these lenses have accelerated since the turn of the 21st century…”
3. Introduction – 2 ^nd^ paragraph: please change “helps” to “help” in the following sentence “…helps support and maintain the integrity of the ocular surface,…”
4. Mid haptic compression section – page 5: Please change “given” to “giving” and add “the” in “while the lens is being worn” in the following sentence “Excess lens pressure on the conjunctival vessels will cause blockage in the blood flow, given the appearance of blanched or whitened focal areas underneath the lens haptic, while lens is being worn, a phenomenon that is usually termed as “blanching”
5. Discussion – 1 ^st^ paragraph: please replace “reduced” with “decreased” in the following sentence “The rate of complications after scleral lens wear has reduced over the years…”
6. Discussion – 2 ^nd^ paragraph: Please add “A” in the beginning and delete “have” in the following sentence “Report by Compan and colleagues have showed the difference in corneal swelling in mini scleral lens user (15.5 mm) having a shallower…”
7. Discussion – 2 ^nd^ paragraph: Please replace “fall” with “falls” and “condition with “conditions” in the following sentence “Although the magnitude of swelling found in these studies fall within the physiological limit in the presence of closed eye condition…”
8. Discussion – 5 ^th^ paragraph: Please replace “loose” with “loosely” and “tight” with “tightly” in the following sentence “While a loose fit lens will show sign of areas of the lens elevated from the ocular surface, a tight fit lens would show indentation…”
9. Discussion – 5 ^th^ paragraph: Consider changing “Whereas” to “Moreover” in the following sentence “Whereas, a significant edge lift would result in the post lens fluid…”
10. Discussion – 5 ^th^ paragraph: Consider replacing the “;” with “,” and adding “it” prior to “can” in the following sentence “…underneath the lens; can potentially reduce the average wearing time of the lens usage,…”
11. Discussion – 6 ^th^ paragraph: Please replace “recommend” with “recommending” and “suggest” with “suggests” in the following sentence “…in addition to recommend the “optimal” lens fitting characteristic, this report also suggest an “acceptable” territory.”

Is the work clearly and accurately presented and does it cite the current literature?

Yes

If applicable, is the statistical analysis and its interpretation appropriate?

Not applicable

Are all the source data underlying the results available to ensure full reproducibility?

Yes

Is the study design appropriate and is the work technically sound?

Yes

Are the conclusions drawn adequately supported by the results?

Yes

Are sufficient details of methods and analysis provided to allow replication by others?

Yes

Reviewer Expertise:

Cornea, Ocular Surface, Cornea Transplantation, Conjunctival Tumors, Cataract Surgery

I confirm that I have read this submission and believe that I have an appropriate level of expertise to confirm that it is of an acceptable scientific standard.

# Response

**Published:** 2022-02-18

**Authors:** Sayan Basu

**Affiliations:** L V Prasad Eye Institute, Hyderabad, India

**Comment 1**: Introduction – 1st paragraph: please change “its” to “their” in the following sentence “Since inception, scleral lenses have undergone drastic changes in its material as well as design.”

***Response***: Thank you for your suggestion. The change has been made in the manuscript.

**Comment 2**: Introduction – 1st paragraph: please change “have” to “has” in the following sentence “However, the popularity of these lenses have accelerated since the turn of the 21st century…”.

***Response***: Thank you for your suggestion. The change has been made in the manuscript.

**Comment 3**: Introduction – 2nd paragraph: please change “helps” to “help” in the following sentence “…helps support and maintain the integrity of the ocular surface,…”.

***Response***: Thank you for your suggestion. The change has been made in the manuscript.

**Comment 4**: Mid haptic compression section – page 5: Please change “given” to “giving” and add “the” in “while the lens is being worn” in the following sentence “Excess lens pressure on the conjunctival vessels will cause blockage in the blood flow, given the appearance of blanched or whitened focal areas underneath the lens haptic, while lens is being worn, a phenomenon that is usually termed as “blanching”.

***Response***: Thank you for your suggestion. The change has been made in the manuscript.

**Comment 5**: Discussion – 1st paragraph: please replace “reduced” with “decreased” in the following sentence “The rate of complications after scleral lens wear has reduced over the years…”.

***Response***: Thank you for your suggestion. The change has been made in the manuscript.

**Comment 6**: Discussion – 2nd paragraph: Please add “A” in the beginning and delete “have” in the following sentence “Report by Compan and colleagues have showed the difference in corneal swelling in mini scleral lens user (15.5 mm) having a shallower…”.

***Response***: Thank you for your suggestion. The change has been made in the manuscript.

**Comment 7**: Discussion – 2nd paragraph: Please replace “fall” with “falls” and “condition with “conditions” in the following sentence “Although the magnitude of swelling found in these studies fall within the physiological limit in the presence of closed eye condition…”.

***Response***: Thank you for your suggestion. The change has been made in the manuscript.

**Comment 8**: Discussion – 5th paragraph: Please replace “loose” with “loosely” and “tight” with “tightly” in the following sentence “While a loose fit lens will show sign of areas of the lens elevated from the ocular surface, a tight fit lens would show indentation…”.

***Response***: Thank you for your suggestion. The change has been made in the manuscript.

**Comment 9**: Discussion – 5th paragraph: Consider changing “Whereas” to “Moreover” in the following sentence “Whereas, a significant edge lift would result in the post lens fluid…”.

***Response***: Thank you for your suggestion. The change has been made in the manuscript.

**Comment 10**: Discussion – 5th paragraph: Consider replacing the “;” with “,” and adding “it” prior to “can” in the following sentence “…underneath the lens; can potentially reduce the average wearing time of the lens usage,…”.

***Response***: Thank you for your suggestion. The change has been made in the manuscript.

**Comment 11**: Discussion – 6th paragraph: Please replace “recommend” with “recommending” and “suggest” with “suggests” in the following sentence “…in addition to recommend the “optimal” lens fitting characteristic, this report also suggest an “acceptable” territory.”

***Response***: Thank you for your suggestion. The change has been made in the manuscript.
